# Documentation of individualized preoperative risk assessment: a multi-center study

**DOI:** 10.1186/s13741-020-00156-2

**Published:** 2020-09-21

**Authors:** Joshua A. Bloomstone, Benjamin T. Houseman, Evora Vicents Sande, Ann Brantley, Jessica Curran, Gerald A. Maccioli, Tania Haddad, James Steinshouer, David Walker, Ramani Moonesinghe

**Affiliations:** 1 Envision Physician Services, 7700 West Sunrise BLVD, Plantation, FL 33322 USA; 2 Department of Anesthesiology, University of Arizona College of Medicine-Phoenix, Phoenix, AZ USA; 3 Centre for Perioperative Medicine, Division of Surgery and Interventional Sciences, University College London, London, UK; 4 Outcomes Research Consortium, Cleveland, OH USA; 5 Health Catalyst, Salt Lake City, UT USA; 6 Quick’rCare, Miami, FL USA; 7 Banner Health, Phoenix, AZ USA

**Keywords:** Individual risk assessment, Surgical risk assessment, Perioperative risk assessment, Risk score, Risk tool, Population risk, Individual risk

## Abstract

**Background:**

Individual surgical risk assessment (ISRA) enhances patient care experience and outcomes by informing shared decision-making, strengthening the consent process, and supporting clinical management. Neither the use of individual pre-surgical risk assessment tools nor the rate of individual risk assessment documentation is known. The primary endpoint of this study was to determine the rate of physician documented ISRAs, with or without a named ISRA tool, within the records of patients with poor outcomes. Secondary endpoints of this work included the effects of age, sex, race, ASA class, and time and type of surgery on the rate of documented presurgical risk.

**Methods:**

The records of non-obstetric surgical patients within 22 community-based private hospitals in Arizona, Colorado, Nebraska, Nevada, and Wyoming, between January 1 and December 31, 2017, were evaluated. A two-sample proportion test was used to identify the difference between surgical documentation and anesthesiology documentation of risk. Logistic regression was used to analyze both individual and group effects associated with secondary endpoints.

**Results:**

Seven hundred fifty-six of 140,756 inpatient charts met inclusion criteria (0.54%, 95% CI 0.50 to 0.58%). ISRAs were documented by 16.08% of surgeons and 4.76% of anesthesiologists (*p* < 0.0001, 95% CI −0.002 to 0.228). Cardiac surgeons documented ISRAs more frequently than non-cardiac surgeons (25.87% vs 16.15%) [*p* = 0.0086, *R*-squared = 0.970%]. Elective surgical patients were more likely than emergency surgical patients (19.57 vs 12.03%) to have risk documented (*p* = 0.023, *R*-squared = 0.730%). Patients over the age of 65 were more likely than patients under the age of 65 to have ISRA documentation (20.31 vs 14.61%) [*p* = 0.043, *R*-squared = 0.580%]. Only 10 of 756 (1.3%) records included documentation of a named ISRA tool.

**Conclusions:**

The observed rate of documented ISRA in our sample was extremely low. Surgeons were more likely than anesthesiologists to document ISRA. As these individualized risk assessment discussions form the bedrock of perioperative informed consent, the rate and quality of risk documentation must be improved.

## Background

According to the 2010 Salzburg statement on shared decision-making, authored by 58 individuals from 18 different countries, patients have the right to be made aware of specific risks regarding their procedure and physicians must “provide accurate information about options and the uncertainties, benefits, and harms of treatment” (BMJ, 2011). As such, individualized surgical risk assessments represent a National Quality Strategy surgical measure and are required by both medical societies and regulatory bodies (American Society of Anesthesiology, 2019; American College of Surgeons, 2019; American Medical Association Measure#358, 2019). These assessments provide additional value in their ability to guide preoperative medical optimization (Stierer & Collop, 2015), surgical and anesthetic management (Chand et al., 2007), and postoperative disposition (Chan et al., 2018). One recent study has shown that such assessments may also enhance patient outcomes (Broughton et al., 2017).

Several validated individualized surgical risk assessment (ISRA) tools are readily available to assist physicians in accurately relaying surgical risk information to patients. These include SORT (Wong et al., 2017), POSSUM (Copeland et al., 1991), p-POSSUM (Prytherch et al., 1998), and the American College of Surgeons (ACS) risk calculator (Bilimoria et al., 2013). Of these, only the ACS tool has been validated for use in the USA. The ACS risk model incorporates 20 “patient predictors” (e.g., age, ASA-PS, BMI, ventilator dependence, diabetes) and the surgical procedure to be performed (CPT code). The current calculator is based on a multivariate analysis of 4.3 million surgical episodes that have been entered in the National Surgical Quality Improvement (NSQIP) database between 2013 and 2017, and provides patient-specific risk for 18 different potential outcomes that could occur within 30 days following one of 1557 surgical procedures (Cohen et al., 2017; American College of Surgeons National Surgical Quality Improvement Program, 2019).

Despite an abundance of perioperative literature that focuses on surgical risk assessment in the USA, it is unknown how often validated preoperative ISRA tools are used or how often individualized risk assessments are documented by perioperative physicians. Given the importance of individual surgical risk assessment, we undertook a 22-medical center, 5-state study of surgical patients with poor outcomes to determine the rate of documentation of individualized preoperative risk assessments, with or without a named ISRA tool, authored by surgeons and anesthesiologists. Secondary endpoints included the effects of age, sex, race, ASA-PS class, and time and type of surgery on the rate of documented individualized risk assessment.

## Methods

### Study design

We carried out a retrospective multi-center study aimed at determining the rate of physician documented individual presurgical risk assessments within the records of patients with poor outcomes. Secondary endpoints included the effects of age, sex, race, ASA class, and time and type of surgery on the rate of documented presurgical risk.

### Participants

Following the Institutional Review Board waiver, all non-obstetric surgical patients cared for within 22 community based, private medical centers located in Arizona, Colorado, Nebraska, Nevada, and Wyoming, between January 1, 2017, and December 31, 2017, were included. A cohort of patients was selected for chart evaluation based on having suffered myocardial infarction, respiratory complications, stroke, acute kidney injury, or death either intraoperatively, within 48 h of surgery, or greater than 48 h after surgery. These intraoperative and postoperative complications were identified from medical records (Cerner) and billing data (MedSeries4) associated with each patient’s surgical episode of care using the following International Classification of Diseases, tenth edition (ICD 10) codes: Z53.8, G97.81, G97.82, I24.9, I46.9, I97.710, I97.711, I97.120, I97.121, J95.4, J95.89, J95.88, T14.8XXA, T14.8XXD, T14.8XXS, I97.820, I97.821, G58.8, J69.8, N99.89, N99.0 (Table 2).

### Analysis

Our study cohort, which included only patients who experienced one or more complications listed above, had their records assessed by a team of clinicians for the presence of an individualized qualitative or quantitative presurgical risk assessment. Study clinicians were trained to assess both anesthesia and surgical preoperative notes for the presence of specific keywords and phrases related to risk assessment, such as “increased risk for acute kidney injury” or “the patient is at increased risk for postsurgical respiratory failure requiring mechanical ventilation.” Clinicians also examined records for the presence of named risk assessment tools, such as “ACS Risk Calculator” or “STS Score.” Standardized electronic health record generated sentences such as “All risks and benefits have been addressed,” were not considered risk assessments. When questions arose during the chart audit process, both the principal investigator and a second auditor reviewed the material to achieve consensus with the primary reviewer.

The primary endpoint of this study was the rate of documented presurgical individualized risk assessments, with or without a named ISRA tool, by anesthesiologists and surgeons. Summary statistics for patient demographics were compiled. The proportion of risk assessments was calculated by assessment type (qualitative, quantitative, and quantitative with a named tool) and note type (surgeon or anesthesiologist). A two-sample proportion test was used to identify whether any difference existed between surgical documentation and anesthesiology documentation of risk. Comparisons of cases with any documented risk assessment were made by age, gender, ASA-PS class, race, time of surgery, emergency status, and case type (cardiovascular versus non-cardiovascular) using a logistic regression model. Mortality by presence of any documented risk assessment was also compared using logistic regression.

## Results

Seven hundred fifty-six out of 140,756 non-obstetric surgical inpatients cared for between January 1, 2017, and December 31, 2017, met inclusion criteria (0.54%, 95% CI 0.50% to 0.58%). The average patient age was sixty-five. Fifty-one percent of patients were male, 89% were Caucasian, 69% were hypertensive, and 36% were diabetic requiring medication. The majority of patients were ASA-PS 3 (43.25%) and ASA-PS 4 (39.68%). Patient demographics and clinical characteristics are shown in Table 1. Respiratory (43.65%), cardiac (39.63%), and renal (9.5%) complications accounted for the majority of organ-specific postsurgical morbidities (Table 2). Two hundred thirteen (28.17%) patients within this cohort died. Of these, 16 (7.51%) patients died intraoperatively, 47 (22.10%) within 48 h of surgery, 103 (48.36%) at greater than 48 h. For 51 patients (22.03%), the time of death could not be determined from the medical record. Out of the total population, 308 (0.22%) patients suffered perioperative cardiac arrest, of which 151 (49%) died. Intraoperative cardiac arrest occurred in 47 (0.03%) patients, 92% of which were ASA-PS classification 3 or higher. Of those with intraoperative cardiac arrest, 24 (51.1%) died: 16 (66.67%) intraoperatively, 3 (12.5%) within 48 h, and 5 (20.83%) at greater than 48 h. Emergency surgical admissions accounted for 158 (21%) patients. Of these patients, 145 (91.8%) were ASA-PS 3 or higher and 43 (27%) died.

**Table 1:** Patient demographics, co-morbidities, and ASA-PS score

	*n* = 756
**Patient characteristic, no. (%)**	
Male	384 (51)
Female	372 (49)
Non-Caucasian	87 (12)
Current smoker within 1 year	147 (19)
Age mean [SD]	65 [17]
**Clinical characteristic, no. (%)**	
Chronic steroid use	55 (7)
Preop ascites	15 (2)
Disseminated cancer	46 (6)
Diabetes	275 (36)
HTN requiring meds	523 (69)
Severe COPD	107 (14)
Acute renal failure	125 (17)
Ventilator dependent	43 (6)
CHF 30 days prior to surgery	91 (12)
Dialysis 14 days prior to surgery	56 (7)
Sepsis within 48 h of surgery	89 (12)
**ASA class, no. (%)**	
Class 1	10 (1)
Class 2	91 (12)
Class 3	327 (43)
Class 4	300 (40)
Class 5	27 (4)
Class 6	1 (0.1)
Emergent	154 (20)

**Table 2:** Complication distribution by ICD-10 code

ICD10	Description	Count	% of total
J9589	Other postprocedural complications and disorders of respiratory system, not elsewhere classified	326	43.12%
I469	Cardiac arrest, cause unspecified	268	35.45%
N990	Postprocedural (acute) (chronic) kidney failure	72	9.52%
I97711	Intraoperative cardiac arrest during other surgery	26	3.44%
I97821	Postprocedural cerebrovascular infarction following other surgery	26	3.44%
I97820	Postprocedural cerebrovascular infarction following cardiac surgery	14	1.85%
I97120	Postprocedural cardiac arrest following cardiac surgery	11	1.46%
I97710	Intraoperative cardiac arrest during cardiac surgery	9	1.19%
J9588	Other intraoperative complications of respiratory system, not elsewhere classified	4	0.53%
**Total**		**756**	

According to the results of our two-sample proportion test (Table 3), surgeons (16.08%) were more likely to document ISRAs than anesthesiologists (4.76%) [*p* value < 0.0001, 95% CI −0.002 to 0.228]. Cardiac surgeons (25.87%) documented ISRAs more frequently than non-cardiac surgeons (16.15%) [*p* value = 0.009, *R*-squared = 0.97%]. The odds of documenting ISRAs for cardiovascular procedures were 1.812 times higher than those for non-cardiovascular procedures (95% CI 1.177 to 2.791).

**Table 3:** Documentation of risk as a function of gender, race, time of surgery, emergency status, ASA score, age, surgical type, mortality, and author of note

	*n*	Any risk assessment	Any risk assessment (%)	*p* value	*R*-squared logistic regression (%)	Odds ratio	CI lower 95%	CI upper 95%
Women	372	71	19.09	0.440	0.080	1.158	0.798	1.678
Men	384	65	16.93			0.864	0.596	1.253
Caucasian	669	114	17.04	0.070	0.460	0.607	0.360	1.025
Non-Caucasian	87	22	25.29			1.648	0.9760	2.782
AM surgery	687	128	18.63	0.128	0.330	1.746	0.815	3.739
PM surgery	69	8	11.59			0.573	0.267	1.227
Emergent	158	19	12.03	0.023^a^	0.730	0.562	0.334	0.945
Non-emergent	598	117	19.57			1.780	1.058	2.994
ASA classes 1/2	101	18	17.82	0.987	0.000	0.995	0.576	1.721
ASA classes 3/4/5	654	117	17.89			1.005	0.581	1.737
Age < 65	308	45	14.61	0.043^a^	0.580	0.671	0.454	0.993
Age 65+	448	91	20.31			1.490	1.008	2.203
Non-cardiovascular procedures	613	99	16.15	0.009^a^	0.970	0.552	0.358	0.850
Cardiovascular procedures	143	37	25.87			1.812	1.177	2.791
Surgeon note	659	106	16.08	< 0.0001^a^			−0.002	0.228
Anesthesiologist note	756	36	4.76					

^a^Statistically significant

Elective surgical patients (19.57%) received ISRA documentation more frequently than emergency surgery patients (12.03%) [*p* value = 0.023, *R*-squared = 0.730%]. In turn, ISRA documentation was 1.78 times more likely to occur in patients who elected to have a surgery than those who had an emergency surgery (95% CI 1.058 to 2.994).

ISRAs were documented more frequently for patients 65 years and older (20.31%) than for patients under 65 (14.61%) [P-value = 0.043, R-squared = 0.580%]. The odds of documenting ISRAs for patients 65 and over is 1.490 times higher than those for patients younger than 65 (95% CI 1.008 to 2.203).

Neither female (19.09%) nor male (16.93%) gender had a statistically significant effect on ISRA documentation [P-value = 0.440, R-squared = 0.080%]. However, the odds of ISRA documentation occurring were 1.158 times higher for female patients than for male patients (95% CI 0.798 to 1.679).

ISRA documentation was not statistically different between non-Caucasians (25.29%) and Caucasians (17.04%) [*p* value = 0.070, *R*-squared = 0.460%]. However, the odds of ISRA documentation occurring were 1.648 times higher for non-Caucasian patients than for Caucasian patients (95% CI 0.976 to 2.782).

No associations with individual risk documentation were found between ASA-PS 1 and 2 (17.82%) and ASA-PS 3, 4, and 5 (17.89%) [*p* value = 0.987, *R*-squared = 0%]. The odds of documenting ISRAs were only 1.005 times higher for patients with ASA-PS 3, 4, and 5 than for patients with ASA-PS 1 and 2 (95% CI 0.581 to 1.737).

No significant relationship [*p* value = 0.128, *R*-squared = 0.330%] existed between ISRA documentation and time of surgery (daytime surgery 18.63%; evening surgery 11.59%). The odds of documenting ISRAs were 1.7460 times higher for patients who had daytime surgery than for those who had surgery in the evening (95% CI 0.815 to 3.739).

There was no significant difference between documentation of preoperative ISRA for patients who survived (17.13%) versus those who died (20.19%) [*p* value = 0.329, *R*-squared = 0.130%]. However, the odds of documenting ISRAs for patients who died were 1.224 higher than for those who survived surgery (95% CI 0.819 to 1.830).

### Group effect model design

A logistic regression model was created to analyze the group effect of age, emergency status, race, and cardiac surgery on the likelihood of ISRA documentation. As a group, these predictors had a significant effect on documenting ISRAs (*p* value = 0.0004) and explain more variability than each measure individually (*R*-squared = 2.86%). The model positively classifies 82.01% of the records.

### Parameters estimates

According to our model, 45.2% of the patients who had documented ISRAs were 65 years and older, non-Caucasian, elected to have a surgery, and underwent cardiovascular surgery.

### Effect likelihood ratio test

Although race did not have a significant relationship upon the documentation of ISRAs (*p* value = 0.070), it was added into the group of predictors because the probability of being a non-Caucasian and having documented ISRAs was 25.29%. According to our model, the individual effect of race (*p* value = 0.0140), age (*p* value = 0.028), emergency status (*p* value = 0.039), and cardiovascular surgery (*p* value = 0.014) were significant.

## Discussion

The primary endpoint of our study was to benchmark the rate of documented individual surgical risk assessments by anesthesiologists and surgeons in a large, multi-state health system. While this study is the first of its kind in the USA, one audit of 496 high-risk surgical patients in the UK demonstrated that only 34 (7%) of preoperative notes included documented risk of organ-specific morbidity or mortality (National Confidential Enquiry into Patient Outcome and Death, 2011). Perioperative complications are said to occur in up to 1:5 surgical patients (Ghaferi et al., 2009; Khuri et al., 2005; Pucher et al., 2014), yet our morbidity rate was far lower (0.54%), likely due to the fact that our patient selection was limited to those who suffered only serious complications, such as perioperative stroke, myocardial infarction, acute respiratory failure, and acute renal failure. Of interest, the rate of cardiac arrest in our study (22:10,000) was much higher than the 5.6:10,000 reported by the National Anesthesia Clinical Outcomes Registry (NACOR). We believe this difference exists because NACOR only includes cardiac arrests that happen within a narrow perioperative window, where as we counted all cardiac arrests from the time of anesthetic induction to the end of hospital stay. Indeed, our rate of perioperative cardiac arrest is in line with several published studies (Nair et al., 2016).

Although surgeons documented individual morbidity and mortality risk more frequently than anesthesiologists, the overall rate for both was surprisingly low. Patients undergoing cardiac surgery were more likely to have documented preoperative risk assessments than patients undergoing non-cardiac surgery. This difference is not simply because surgeons have classically borne the responsibility of assisting patients with weighing the risk of surgery, as this would apply to both cardiac and non-cardiac surgeons (Hanson & Pitt, 2017). We note that cardiac surgeons have had powerful individualized risk assessment tools, such as the Society of Thoracic Surgeons (STS) risk calculator, at their disposal for almost 40 years (Cornelissen & Arrowsmith, 2006). The importance of IRSA in cardiac surgery’s culture may thus explain the difference.

We were surprised to find that both surgeons and anesthesiologists were more likely to document individual risk in patients undergoing elective surgery rather than emergency surgery, given that patients undergoing emergency surgery are at substantially higher risk for both intraoperative and postsurgical adverse events (Ingraham et al., 2011). Indeed, emergency surgical patients accounted for 21% of our cohort and 34% of its deaths. This mortality rate is substantially higher than published emergency surgical mortality (Mullen et al., 2017), and is perhaps due to the fact that our cohort had a higher than average number of older patients with higher ASA-PS.

We were also surprised to find that patients with ASA-PS scores of 3,4, or 5 did not have higher levels of ISRA documentation than ASA-PS scores of 1 or 2. Although ASA-PS is a population-based risk assessment tool that lacks the ability to provide individualized organ-specific morbidity or mortality risk (Moonesinghe et al., 2013), it has been validated as a reliable predictor of postsurgical morbidity and mortality (Hackett et al., 2018). We did not differentiate between ASA-PS 3, ASA-PS 4, and ASA-PS 5 in this study. We also acknowledge that there can be significant inter-anesthesiologist variability when assigning ASA-PS scores (Ranta et al., 1997; Haynes & Lawler, 1995; Owens et al., 1978).

Individual risk assessments were documented more frequently in patients older than 65 and in non-Caucasians, which may relate to greater pre-existing chronic conditions in these patient groups (National Academies of Sciences, Engineering, and Medicine; Health and Medicine Division, et al., 2017; Boddaert et al., 2014). The lack of correlation between perioperative death and documented risk assessment is astonishing, given that perioperative mortality is generally neither a surprise to the surgeon nor the anesthesiologist (White, 2003). To this point, the 2000 Report of the National Confidential Enquiry into Perioperative Deaths found that only 12 in 100 deaths were not expected (National Confidential Enquiry into Perioperative Deaths, 2019). Finally, while all anesthesiologists in our study documented an ASA-PS, only 1 in 20 (5%) documented the risk of either individual organ system morbidity or mortality.

According to CMS conditions of participation, a surgical patient must be examined by a physician immediately prior to surgery to evaluate the risk of anesthesia and of the procedure to be performed. Additionally, this assessment must be “specific to each patient” (Center for Medicare and Medicaid Services, 2013). Unfortunately, the ASA-PS score does not provide an assessment of individualized risk, it provides a population-based risk assessment and, while it is a useful *general* predictor of morbidity and mortality (Sankar et al., 2015), it lacks the ability to specifically predict organ system morbidity or mortality, and was not originally intended to be used as a measure to predict operative risk (Minto & Biccard, 2014; Doyle & Garmon, 2019). It is thus surprising that the ASA-PS remains the standard for surgical risk assessment given the availability of validated, individualized risk assessment tools that provide organ-specific morbidity risk in addition to the risk of 30-day mortality. Unfortunately, knowledge of individual surgical risk assessment tools remains low and, as we have demonstrated, utilization is sparse. Sadly, only 1.3% (10) of these 756 patients had morbidity or mortality risk documented alongside a named risk assessment tool, such as the ACS risk calculator (Table 4). Given that the calculator is easy to access and use, and that it is available at no charge makes these findings even more surprising. Also, surprising is the continued endorsement of a population-based risk assessment tool that neither incorporates procedure type nor provides organ-specific risk. The use of the ASA-PS score as a method to enhance shared decision-making and informed consent is not in line with the aforementioned Salzburg statement, which makes clear the right of patients to be made aware of specific risks regarding their procedure. Additionally, because the ASA-PS score does not inform clinicians about organ-specific risk, appropriate, targeted preoperative medical optimization, and perioperative medical management remains uninformed. Though our study was not designed to demonstrate that preoperative risk assessment improves outcomes, prospective surgical risk assessment has been shown to decrease mortality in patients undergoing both elective (NCEPOD, 2011) and emergency surgery (Poulton et al., 2019). Whether the cause of reduced mortality is due to better informed perioperative medical management or patients deciding not to have surgery following informed shared decision-making remains unclear (Hall et al., 2012).

**Table 4:** Use of named risk assessment tools in 756 surgical patient charts

Risk assessment tool name	Anesthesiologist note	Surgeon note	Total
ACS	2	1	3
NSQIP	2	0	2
STS	0	5	5
Total	4	6	10

Our study has several potential limitations. First, the majority of our identified patients were older, hypertensive, diabetic, and either ASA-PS class 3 or 4. It is possible that surgical patients who did not meet criteria for chart selection did indeed have documented risk assessments. However, we hypothesized that if any group of patients were likely to have documented individualized risk assessment, it would be those with co-morbidities, those who underwent emergency surgery, and those who died either intraoperatively or within 48 h of surgery. Rather than a random selection of patients from our 2017 inpatient surgical population, we felt that identifying patients based on serious intraoperative or postoperative complications or death was the best way to identify a cohort of patients most likely to have had documented preoperative risk assessment. We acknowledge that our results may have differed had we sampled a larger number of patient records rather than limiting the study to those records of patients who suffered significant complications. Additionally, because we only included preoperative assessments authored by surgeons and anesthesiologists, it is possible that patients may have been made aware of perioperative risk by other non-perioperative physicians. Additionally, we intentionally disregarded statements, such as “all risks and benefits have been addressed” because these statements are part of our standard electronic preoperative notes. It is certainly possible that either an anesthesiologist and/or surgeon discussed risk with their patient, but rather than document the discussion, chose to use the available prewritten sentence. Finally, to mitigate the risk of bias from local practice patterns, including assignment of ASA-PS scores, we chose to sample a group of patients from 22 different medical centers located in 5 different states.

## Conclusions

Despite being a standard of care, individualized pre-surgical risk assessments are rarely documented by either anesthesiologists or surgeons. Surgeons were more likely than anesthesiologists to document individual morbidity and mortality risk. While ASA-PS score was documented for all patients by anesthesiologists, only 1 in 20 notes authored by anesthesiologists included documentation of organ-specific risk or the risk of mortality. Providing patients with individualized pre-procedural risk assessment benefits patients and their clinicians, represents good perioperative practice, is in line with the Salzburg statement, and is our ethical responsibility. Without documented individualized risk assessment, one must question the quality of informed consent being obtained; after all, this quoted prediction of risk provides the foundation for perioperative decision-making. Without it, are patients able to adequately make a balanced and informed decision whether to undergo surgery?

## Data Availability

The datasets used and/or analyzed during the current study are available from the corresponding author upon reasonable request.

## Abbreviations

ISRA: Individual surgical risk assessment
ASA-PS: American Society of Anesthesiology Physical Status
NACOR: National Anesthesia Clinical Outcomes Registry
ACS: American College of Surgeons
CPT: Current procedural terminology
POSSUM: Physiological and operative severity score for the enumeration of mortality and morbidity
p-POSSUM: Portsmouth-POSSUM
SORT: Surgical outcomes risk tool
